# Effectiveness of Acupuncture on Urinary Retention: A Meta-Analysis

**DOI:** 10.1155/2021/2500282

**Published:** 2021-09-29

**Authors:** Chengwen Zheng, Zaoying Li, Haizhen Lu, Yi Zhou

**Affiliations:** ^1^Basic Medical School of Chengdu University of Traditional Chinese Medicine, Chengdu 610000, China; ^2^Clinical Medical School of Chengdu University of Chinese Medicine, Chengdu 610000, China

## Abstract

**Objectives:**

This study aimed to evaluate the safety and efficacy of acupuncture in the treatment of urinary retention (UR).

**Methods:**

Randomized controlled trials investigating the effectiveness of acupuncture in the treatment of UR were identified by searching seven comprehensive databases (Cochrane Library, PubMed, Embase, China National Knowledge Infrastructure, Wanfang Database, China Science and Technology Journal Database, and Chinese Biomedical Literature Database) prior to September 2020. Data analysis was performed using RevMan, version 5.3, and Stata software, version 14.0.

**Results:**

A total of 12 studies with 979 participants were included. A random-effects model was used to conduct a meta-analysis on the acupuncture group and the control group. The results show that acupuncture can effectively promote spontaneous urination and reduce anxiety in patients with poor urination (relative risk: 1.35; 95% confidence interval (CI): 1.19–1.53; *P* < 0.00001). The random-effects model showed significant differences in residual urine volume between the acupuncture group and the control group (MD: −84.79, 95% CI: −135.62 to −33.94; *P*=0.001).

**Conclusion:**

Acupuncture is safe and effective in the treatment of UR. However, since the current level of evidence is limited, high-quality, large-sample, multi-center, clinical randomized controlled trials are needed to further confirm our conclusions in the future.

## 1. Introduction

Urinary retention (UR), which is characterized by an inability to completely empty the bladder, is a common complication of surgery and anesthesia [1]. However, there is currently no standardized definition of UR. It is generally believed that a remaining urinary volume of >300 ml in the bladder is the diagnostic basis for UR [2, 3]. The incidence of UR is between 5% and 70%, and the risk of UR increases with age, which seriously affects quality of life for patients [4, 5]. However, there are currently no definitive and effective treatments for UR [6–8]. Although bladder function training, physical therapy, surgical treatment, drug treatment, and other methods are widely used in the clinic [9–13], one trial has shown that bladder training by catheter clamping offers no advantage over free-drainage removal of short-term urinary catheters [14]. Catheterization is generally considered the best treatment option for UR, but it increases the risk of urinary tract infection [15]. Although cholinergic drugs and prostaglandins are promising treatments for UR, there is still insufficient evidence to support their use [16].

Acupuncture, which encompasses a wide range of treatments, is recommended in various clinical guidelines for the treatment of multiple diseases, such as dysmenorrhea, rhinitis, and stroke [17–19]. At present, a large number of studies have shown that acupuncture regulates neurotransmitters and can objectively improve urodynamics [20, 21]. However, the European Association of Urology and the American Urological Association have not yet included acupuncture in treatment guidelines [22, 23]. Therefore, we conducted a meta-analysis and merged data to provide clinical decision-making recommendations. In this meta-analysis, we identified clinical randomized controlled trials on acupuncture and moxibustion for UR and distinguished the commonly used acupuncture point selection schemes to clarify the curative effect of acupuncture on UR.

## 2. Methods

The systematic review protocol was developed using guidance from the Preferred Reporting Items for Systematic Reviews and Meta-Analyses (PRISMA) statement and is registered in PROSPERO (CRD42021228237).

### 2.1. Search Strategy

Randomized controlled trials were retrieved by searching the following databases from the date of inception to September 2020: Cochrane Library, PubMed, Embase, China National Knowledge Infrastructure, Wanfang Database, China Science and Technology Journal Database, and Chinese Biomedical Literature Database. No language restrictions were applied. According to the characteristics of each database, the search terms were as follows: (“acupuncture” OR “electro-acupuncture” OR “acupuncture points” OR “moxibustion”) AND (“urinary retention” OR “post‐operative complications” OR “urination disorders”).  Table 1 shows the PubMed search strategy as an example.

This search strategy was modified as required for other electronic databases.

### 2.2. Inclusion and Exclusion Criteria

#### 2.2.1. Study Types

Only randomized controlled trials were included, regardless of blinding. Studies were written in Chinese or English.

#### 2.2.2. Participants

Participants were patients with clinically confirmed UR, regardless of type, sex, age, and country.

#### 2.2.3. Intervention

The experimental group underwent several types of acupuncture therapy, including electric, auricular, manual, and scalp acupuncture. Moxibustion, warm needle, fire needle, and acupoint injections were excluded. The control group received no intervention, placebo, drug therapy, rehabilitation training, sham acupuncture, or other conservative treatment, such as urinary catheterization. Studies comparing acupuncture plus traditional Chinese medicine with traditional Chinese medicine, as well as those comparing acupuncture plus moxibustion with moxibustion, were excluded.

#### 2.2.4. Outcomes

The main outcome indicators were the change in the number of voluntary urinations and the change in residual urine volume (RUV).

### 2.3. Study Selection

All studies were imported into NoteExpress, and two reviewers (Chengwen Zheng and Zaoying Li) independently screened them. After reading the titles and abstracts, duplicate articles and articles that did not meet the inclusion criteria were excluded. Studies that met the aforementioned predetermined inclusion criteria were included. Any inconsistency in the data extracted was resolved by a third reviewer (Haizhen Lu). A PRISMA flow diagram was used to describe the process of study selection.

### 2.4. Data Extraction

Data extraction was performed independently by two reviewers (Chengwen Zheng and Zaoying Li). After comparing the results and verifying the original documents, the authenticity and completeness of all data were finally determined. The extracted content included author, publication year, sample size, male to female ratio, age of subjects, method used in the study, intervention measures used in each group, evaluation times of outcome indicators, evaluated outcome indicators, and each acupuncture point selected by the research institute. All disagreements were resolved by consensus between the two initial reviewers and the third reviewer (Haizhen Lu).

### 2.5. Risk of Bias in Individual Studies

Two reviewers (Chengwen Zheng and Zaoying Li) assessed the risk of bias in included studies using the Cochrane Collaboration's risk-of-bias tool. Each study was classified as having low, unclean, or high-risk bias based on seven items: (1) random sequence generation; (2) allocation concealment; (3) blinding of participants and personnel; (4) blinding of outcome assessment; (5) incomplete outcome data; (6) selective reporting; and (7) other. Any disagreements were resolved by the third reviewer (Haizhen Lu). The risk-of-bias assessment was summarized using Review Manager 5.3.

### 2.6. Data Analysis

A quantitative analysis was performed using Cochrane Collaboration software (RevMan, version 5.3.5) for the meta-analysis and using Stata software version 14.0 for funnel plot analysis. The relative risk (RR) with 95% confidence interval (CI) was selected as the statistic for dichotomous data. Because the unit of the outcome indicator is consistent, the mean difference (MD) with 95% CI is used to describe continuous variables. The random-effects model was used to analyze all data. During the heterogeneity test, the chi-square test was performed first, and based on its findings, estimates of heterogeneity (𝐼^2^) were applied. If heterogeneity was high, the source of heterogeneity was explored. A subgroup or sensitivity analysis was performed to investigate the stability of the meta-analysis. Publication bias was explored using a funnel plot analysis.

## 3. Results

### 3.1. Study Selection

**Figure 1:**
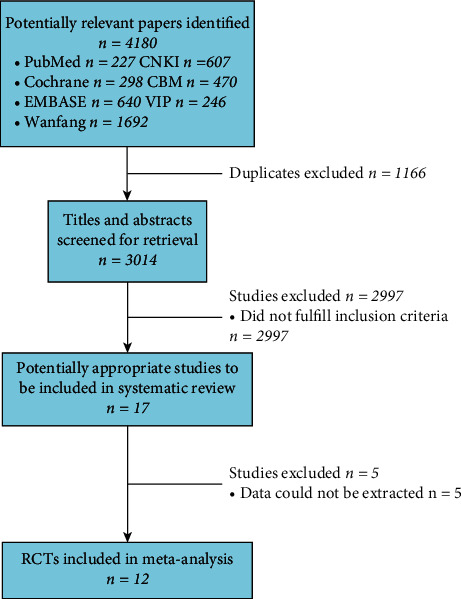
PRISMA flow diagram.

A total of 4,180 unique studies were identified using the search strategy. After duplicate studies were excluded, 3,014 related studies were screened out. After reading the titles and abstracts of these studies, 17 related studies were identified. Further screening for eligibility was performed by two independent reviewers according to the inclusion and exclusion criteria. Eventually, 12 studies were included in the meta-analysis (Figure 1).

### 3.2. Study Characteristics

A total of 12 randomized controlled trials [25–36] were included in this study, with a total of 979 patients with UR (483 in the experimental group and 496 in the control group). All participants were Chinese. The intervention measure in the experimental group was electric acupuncture or manual acupuncture. In the control group, seven groups [25, 26, 28, 30–33] received drug intervention, of which six groups [25, 26, 30–33] received intramuscular injection of neostigmine, and one group [28] received oral Qianlieantong and finasteride tablets. The remaining patients in the control group were treated with conventional symptomatic treatment. All trials report on spontaneous urination, while three trials [28, 29, 31] report changes in RUV. The characteristics of included studies are presented in Table 2.

### 3.3. Risk of Bias in Included Studies

**Figure 2:**
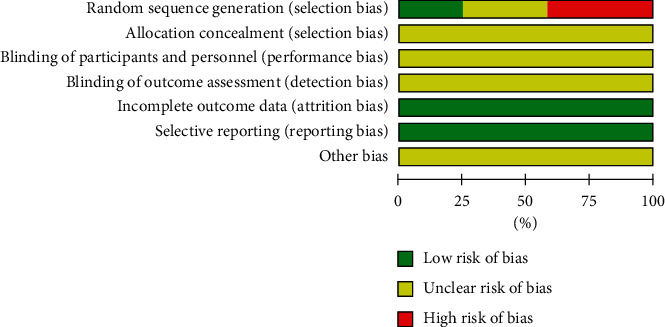
Diagram of the risk of bias.

**Figure 3:**
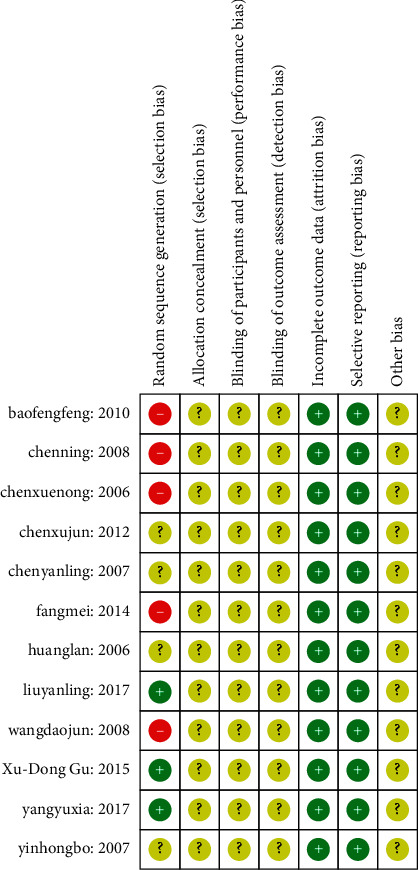
Summarized risk of bias.

The 12 papers included in this study were all randomized controlled trials. Four studies [25, 26, 30, 34] did not report the specific method of random generation, while 5 studies [27, 31–33, 35] used random methods with a higher risk of bias, such as the order of visits [31, 32], order of consultations [27], surgical duration [33], and time of disease onset [35]. None of the studies mentioned the application of allocation hiding related information. In terms of blinding, none of the studies described patient or evaluator blinding. All included randomized controlled trials had a low risk of bias against incomplete data and selective reporting. The risk of bias assessment is summarized in Figures 2 and 3.

### 3.4. Meta-Analysis Results

#### 3.4.1. Spontaneous Urination

**Figure 4:**
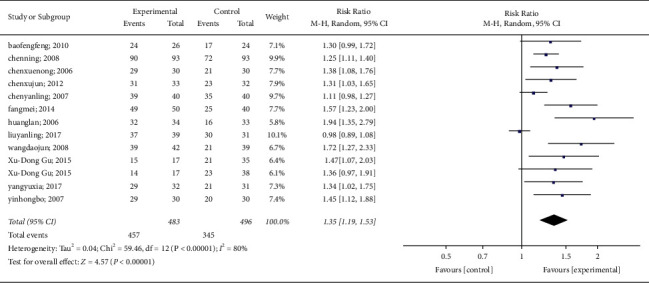
Results of the meta-analysis on spontaneous urination.

All studies reported changes in the number of people with spontaneous urination after acupuncture. Statistics revealed heterogeneity among the 12 trials (*P* < 0.00001, *I*^2^ = 80%). Therefore, a random-effects model was used to conduct a meta-analysis on the acupuncture group and the control group. The results show that acupuncture can effectively promote spontaneous urination and reduce anxiety in patients with poor urination (RR: 1.35; 95% CI: 1.19–1.53; *P* < 0.00001). The results of the meta-analysis are shown in Figure 4.

#### 3.4.2. RUV

**Figure 5:**
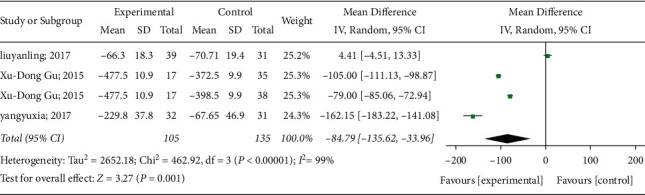
Meta-analysis results of RUV.

Three of the 12 studies reported changes in RUV after acupuncture. Statistics revealed heterogeneity among the three randomized controlled trials (*P* < 0.00001, *I*^2^ = 99%). The random-effects model showed a significant difference in RUV between the acupuncture group and the control group (MD: −84.79, 95% CI: −135.62 to −33.94; *P*=0.001). This indicates that acupuncture can effectively relieve UR. The results of this meta-analysis are shown in Figure 5.

#### 3.4.3. Subgroup Analyses

We divided the included studies into three subgroups according to the interventions used in the experimental group, the interventions used in the control group, and the disease type to discuss the efficacy of acupuncture and study heterogeneity.

**Figure 6:**
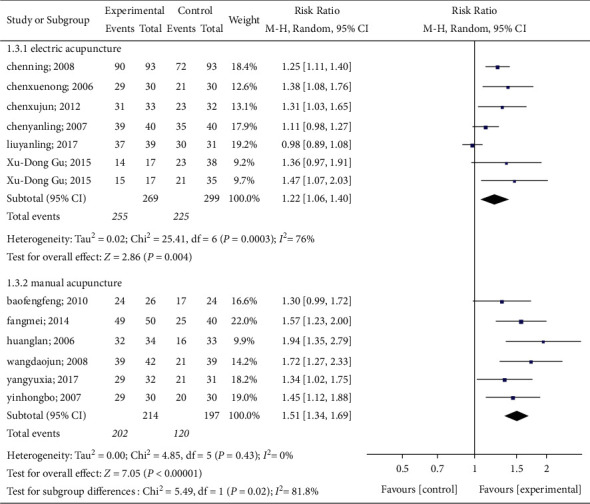
A meta-analysis of the effects of acupuncture type on spontaneous urination.

*(1) Interventions in the Experimental Group*. The acupuncture group was divided into electric acupuncture and manual acupuncture. Six articles used electric acupuncture, and six articles used manual acupuncture. Statistics showed significant heterogeneity in the treatment of UR with electrical acupuncture (*P*=0.0003, *I*^2^ = 76%), and there was a significant difference in the effective rate between the electrical acupuncture group and the control group (*P*=0.004, RR: 1.22, 95% CI: 1.06 to 1.40). Moreover, there was a significant difference in the effective rate between the manual acupuncture group and the control group (*P* < 0.0001, RR: 1.51, 95% CI: 1.34 to 1.69), but there was no significant heterogeneity (*P*=0.43*P* = 0.43, *I*^2^ = 0%). The results of this meta-analysis are shown in Figure 6.

**Figure 7:**
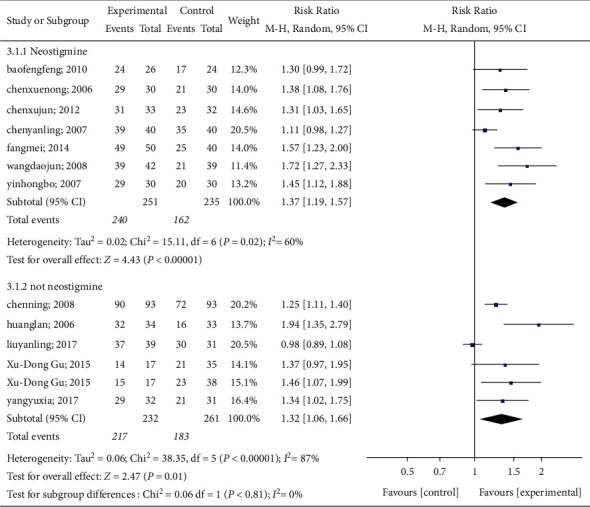
A meta-analysis of the effects of neostigmine on spontaneous urination.

*(2) Interventions in the Control Group*. Based on the interventions in the control group, this group was divided into a neostigmine group and a non-neostigmine group according to whether or not neostigmine was used. Seven of 12 articles used neostigmine in the control group, while the remaining 5 did not. Pooled data show a significant difference in the effective rate between the acupuncture group and the neostigmine group (*P* < 0.00001, RR: 1.37, 95% CI: 1.19 to 1.57) with obvious heterogeneity (*P*=0.02, *I*^2^ = 60%). A significant difference was observed in treatment effectiveness between the acupuncture group and the non-neostigmine control group (*P*=0.01, RR: 1.32, 95% CI: 1.06 to 1.66), and marked heterogeneity was observed (*P* < 0.0001, *I*^2^ = 87%). The results of this meta-analysis are shown in Figure 7.

### 3.5. Publication Bias

**Figure 8:**
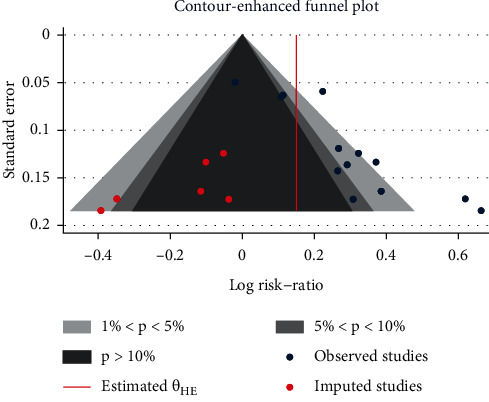
Publication bias and trim-and-fill analysis.

We conducted a funnel plot analysis of the 12 randomized controlled trials to analyze publication bias. The inverted funnel in Figure 8 is asymmetric on the left and right sides. A trim-and-fill analysis found that the results mostly fell in the shadow of *P* < 0.1, indicating that there may be unpublished documents that are not statistically significant, which may lead to publication bias. In addition, since most of the included studies were in Chinese, language bias may be present.

## 4. Discussion

### 4.1. Principal Findings

This study shows that acupuncture has obvious advantages over conventional treatment in improving UR. Acupuncture can promote spontaneous urination to relieve the symptoms of UR and reduce RUV. In terms of safety, no studies mentioned adverse events, so the safety of acupuncture for UR could not be verified. Due to the high heterogeneity in this study, we divided the study into three subgroups according to the intervention used in the experimental group, the intervention used in the control group, and the disease type. The results show that heterogeneity was significantly reduced when the test group was classified by the type of acupuncture; thus, it can be inferred that the type of acupuncture may have been the source of the observed heterogeneity.

### 4.2. Mechanism of Acupuncture

UR usually occurs after childbirth, myelopathy, or lumbar spine surgery. There are many reasons for UR. First, damage to nerves that control the interaction between the bladder and the brain may cause loss of bladder control, leading to UR. Second, dysfunction in muscles or nerves that control urination may affect normal bladder and ureter function, leading to bladder dysfunction or failure of pelvic floor relaxation, resulting in UR [36]. Studies have shown that the direct nerve signal that induces bladder contraction may originate from the Barrington nucleus [37]. The Barrington nucleus is the core component of the urinary circuit. It increases the possibility of urination by activating the spinal excitatory pathway or inhibiting the spinal inhibitory mechanism [38]. In animal experiments, acupuncture changes the discharge characteristics of neurons related to bladder activity in and around the Barrington nucleus, thereby regulating bladder urination [39, 40].

According to traditional Chinese medicine, the kidney governs water balance, which means that metabolism of body fluids depends on the Qi transformation of kidney essence. The kidney has an exterior-to-interior relationship with the bladder, and normal transformation-transportation of kidney and bladder is responsible for urine production and excretion. An abnormal bladder Qi transformation can induce UR, resulting in a low urine volume, dripping out, or even occlusion. Simulating acupoints can unblock the meridians and regulate internal organs. According to basic theories of traditional Chinese medicine, acupuncture can promote Qi transformation of the kidney and bladder and restore bladder function. The effectiveness of acupuncture depends on techniques that are difficult for doctors to master. These techniques include needle insertion angle and depth, as well as the holding force before the needle is removed. Use of different techniques by different practitioners will impact the effects of treatment.

### 4.3. Implications for Clinical Practice and Further Research

This study shows that acupuncture is a safe and convenient treatment method that has a good effect on postpartum and postoperative UR. Frequently used acupuncture points include Ciliao (BL32), Sanyinjiao (SP6), Zhongji (RN3), and Guanyuan (RN4). Further research and screening of fixed and effective acupoints should be carried out to form a fixed acupuncture treatment plan to benefit more patients with UR. High-quality, large-scale, multi-center, clinical randomized controlled studies are required to obtain more accurate analysis results. Further research should adopt strict randomization, allocation concealment, and blinding, as well as clear inclusion and exclusion criteria, and the criteria for curing disease should be standardized and unified.

### 4.4. Advantages of This Research

The current systematic review focused on postpartum UR and drug intervention. No up-to-date systematic reviews on acupuncture interventions have been published in the last two years. Moreover, most previous systematic reviews only studied a single type of UR or a single type of intervention in the control group. As far as we know, this study is the first systematic review and meta-analysis to comprehensively evaluate the effectiveness of acupuncture and moxibustion in the treatment of UR without limiting the type of UR or the type of intervention in the control group. In the present study, the effectiveness of acupuncture in the treatment of UR has been clarified to a certain extent. We mainly focused on the effects of common forms of acupuncture, which reduced the possible variability in other forms of acupuncture and moxibustion and enabled a more accurate assessment of the role of acupuncture in UR. We also screened the most frequently used acupoints from the included literature. We believe the findings of this study will aid future research on acupuncture in the treatment of UR.

### 4.5. Limitations

The limitations of this study are as follows. First, due to lack of high-quality, multi-center, clinical randomized controlled studies, the level of evidence in this study was limited. Second, publication bias exists, and the analysis results are not sufficiently robust. Third, only objective indicators, such as spontaneous urination and RUV, which are closely related to clinical practice, were evaluated. Conversely, indicators that are difficult to systematically divide and that are clinically heterogeneous, such as curative effect grade and patient experience, were not analyzed. Finally, due to lack of reports on the adverse effects of acupuncture, the possible adverse reactions during implementation of acupuncture were not systematically evaluated.

## 5. Conclusion

This meta-analysis of 12 randomized controlled trials shows that acupuncture is safe and effective in the treatment of UR. However, since the current level of evidence is limited, high-quality, large-sample, multi-center, clinical randomized controlled studies are needed to further confirm the conclusions in the future.

## Figures and Tables

**Table 1:** PubMed search strategy.

Number	Search terms
#1	“Acupuncture”[Mesh]
#2	Acupuncture Therapy[Title/Abstract]
#3	acupressure[Title/Abstract]
#4	Electroacupuncture^*∗*^[Title/Abstract]
#5	electro-acupuncture^*∗*^[Title/Abstract]
#6	acupoint^*∗*^[Title/Abstract]
#7	meridian^*∗*^[Title/Abstract]
#8	non‐meridian^*∗*^[Title/Abstract]
#9	trigger^*∗*^[Title/Abstract]
#10	Moxibustion [Title/Abstract]
#11	moxa^*∗*^[Title/Abstract]
#12	Acupuncture points[Title/Abstract]
#13	auriculotherapy [Title/Abstract]
#14	zhenjiu[Title/Abstract] OR zhen jiu [Title/Abstract] OR zhenci[Title/Abstract] OR zhen ci[Title/Abstract] OR cizhen[Title/Abstract] OR dianzhen[Title/Abstract] OR dian zhen[Title/Abstract] OR zhen ya[Title/Abstract] OR er zhen[Title/Abstract] OR ti zhen[Title/Abstract] OR she zhen[Title/Abstract] OR tou pi zhen[Title/Abstract] OR xue wei[Title/Abstract]
#15	(((ching[Title/Abstract]) AND (lo[Title/Abstract])) OR (jing[Title/Abstract] AND Luo[Title/Abstract])) OR (jinglo[Title/Abstract])
#16	#1 or #2 or #3 or #4 or #5 or #6 or #7 or #8 or #9 or #10 or #11 or #12 or #13 or #14 or #15
#17	Urinary Retention[MeSH terms]
#18	post‐operative Complications[Title/Abstract]
#19	Urination Disorders[Title/Abstract]
#20	(urin^*∗*^[Title/Abstract] AND (retent^*∗*^[Title/Abstract] OR retain^*∗*^[Title/Abstract]))
#21	((bladder^*∗*^[Title/Abstract] OR void^*∗*^[Title/Abstract]) AND (retent^*∗*^[Title/Abstract] OR retain^*∗*^[Title/Abstract]))
#22	#17 or #18 or #19 or #20 or #21
#23	#16 and #22

**Table 2:** Characteristics of included studies.

Study	Country	Experimental design	Type of urine retention	Experimental group				Acupoint	Stimulus parameter		
				Sample size	Age (years)	Sex (male/female)	Intervening measure		Stimulus	Time (min)	Frequency (n/day)

Xu-Dong Gu, 2015	China	RCT, 3 arms	SCI induced urinary retention	34	39.6 ± 7.6	1/4	EA + CIC	BL31–34	Pulse frequency of 20 Hz	20	1
Yangyuxia, 2017	China	RCT, 2 arms	Post-stroke urinary retention	32	60 ± 10	20/12	Acupuncture + conventional therapy	Twelve well-points	Shallow insertion	—	1
Baofengfeng, 2010	China	RCT, 2 arms	Postpartum urinary retention	26	21–37^#^	0/26	Acupuncture + TDP	RN3–4, ST36, SP6, KI3, BL22–23, BL39	Acupuncture manipulation	20	1
Chinning, 2008	China	RCT, 2 arms	Urine retention after surgery	93	40.5^*∗*^	27/66	EA + clipping of the urine tube	RN3, SP9, ST36	Patient tolerance	30	1
Chenxujun, 2012	China	RCT, 2 arms	Postpartum urinary retention	33	20–36^#^	0/33	EA	RN3, RN6, ST27–28	2/100 Hz	30	1
Chenxuenong, 2006	China	RCT, 2 arms	Urine storage after cervical cancer surgery	30	35–69^#^	0/30	EA	ST28, BL28, BL32, SP6, SP8	3.3 Hz	30	2
Chenyanling, 2007	China	RCT, 2 arms	Postpartum urinary retention	40	21–34^#^	0/40	EA	RN2–3, SP6, ST36	2 Hz	30	1–2
Fangmei, 2014	China	RCT, 2 arms	Urine retention after hemorrhoid surgery	50	20–60^#^	20/30	Acupuncture	RN3, SP6, SP10	Acupuncture manipulation	15	1
Huanglan, 2006	China	RCT, 2 arms	Urine retention after intraspinal anesthesia	34	—	—	Acupuncture	ST36, SP6, RN3–4	Acupuncture manipulation	15–20	—
Liuyanling, 2017	China	RCT, 2 arms	Prostatic hyperplastic urine retention	39	60 ± 12	39/0	EA	BL23, BL28, BL32–33, RN4, RN6, SP6, SP9, KI10, KI6	Patient tolerance	15–20	1
Wangdaojun, 2008	China	RCT, 2 arms	Postpartum urinary retention	42	18–35^#^	0/42	Acupuncture	PC6, RN4, LI4, LR3, LR10–11, SP6	Acupuncture manipulation	30	2
Yinhongbo, 2007	China	RCT, 2 arms	Postoperative non-obstructive urinary retention	30	16–67^#^	—	Acupuncture	RN3, BL32, ST28, SP6, SP9	Acupuncture manipulation	20	1

Study				Control group				Outcome		Measurement timepoint (days)	
				Sample size	Age (years)	Sex (male/female)	Intervening measure				

Xu-dong Gu, 2015				35	40.6 ± 9.8	1/4	CIC	RUV; number of patients with bladder balance		90	
				38	40.75 ± 12.5	1/3	Sham acupuncture ± CIC				
Yangyuxia, 2017				31	55 ± 11	21/10	Conventional therapy	RUV		20	
Baofengfeng, 2010				24	21–37^#^	0/24	Neostigmine + hot compress of the lower abdomen	RUV		3	
Chinning, 2008				93	42.0^*∗*^	25/68	Clipping of the ureter	Voluntary urination		5	
Chenxujun, 2012				32	20–36^#^	0/32	Neostigmine	Voluntary urination		3 h	
Chenxuenong, 2006				30	35–69^#^	0/30	Neostigmine + conventional therapy	Voluntary urination, RUV		5	
Chenyanling, 2007				40	22–35^#^	0/40	Neostigmine	Voluntary urination		6 h	
Fangmei, 2014				40	20–59^#^	18/22	Neostigmine	Voluntary urination		1 h	
Huanglan, 2006				33	—	—	Conventional therapy	Voluntary urination		—	
Liuyanling, 2017				31	63 ± 11	0/31	Qianlieantong tablets; finasteride tablets	RUV		21	
Wangdaojun, 2008				39	18–35	0/39	Neostigmine	Voluntary urination		1	
Yinhongbo, 2007				30	16–67	—	Neostigmine + conventional therapy	Voluntary urination		5	

Abbreviations: ∗ = average; # = range; CIC = clean intermittent catheterization; residual urine volume (RUV) judging criteria: cure—RUV < 100; valid—100 ≤ RUV < 200; invalid—RUV > 200.

## Supplementary Materials

Supplementary Materials S1: checklist of items to include when reporting a systematic review or meta-analysis (PRISMA).
